# Cell secretion from the adult lamprey supraneural body tissues possesses cytocidal activity against tumor cells

**DOI:** 10.1186/s40064-015-1270-6

**Published:** 2015-10-01

**Authors:** Yue Pang, Shiyue Wang, Wei Ba, Qingwei Li

**Affiliations:** College of Life Science, Liaoning Normal University, Dalian, 116081 China; Lamprey Research Center, Liaoning Normal University, Dalian, 116081 China

## Abstract

The supraneural body was identified in the adult lamprey, and its secretions induced the death of a variety of tumor cells but had no effect on normal cells. The cell secretions from different lamprey tissues were separated, and these secretions killed human tumor cells to varying degrees. The cell secretions induced remarkable cell morphological alterations such as cell blebbing, and the plasma membrane was destroyed by the secretions. In addition, the secretions induced morphological alterations of the mitochondria, cytoskeletal structure, and endoplasmic reticulum, eventually leading to cell death. These observations suggest the presence of a novel protein in the lamprey and the possibility of new applications for the protein in the medical field.

**Electronic supplementary material:**

The online version of this article (doi:10.1186/s40064-015-1270-6) contains supplementary material, which is available to authorized users.

## Background

Bajoghli et al. (2011) identified thymus-like lympho-epithelial structures, termed thymoids, in the tips of gill filaments and neighboring secondary lamellae (both within the gill basket) in lamprey larvae, implicating the thymoid as the development site of T-like cells in lampreys. In lampreys, hematopoietic activity is located in the typhlosole of the intestine in both larval and adult stages. In addition, the kidney and gill regions have been linked to hematopoietic activity (Amemiya et al. 2007). In the adult lamprey, Piavis and Hiatt (1971) confirmed that the fatty tissue rod embedded in the fibro cartilaginous sheath dorsal to the nerve cord is the principal hematopoietic organ. Furthermore, George and Beamish (1974) demonstrated the hemocytology of the supraneural body in the lamprey during several phases of the life cycle. In addition, scientists found that the SB is not only relative to the hematopoietic organ but also comprises immune tissues in the lamprey because numerous immune molecules were identified in the SB of adult lamprey (Amemiya et al. 2007).

As one of the oldest living species on the earth, lamprey surely has evolved some tumor-defense modes, for spontaneous tumors have never been found in lamprey. Nevertheless, it is unclear how the defenses work. Here, we hypothesize the anti-cancer behaviors somehow link with the supraneural body because of its lympho-hematopoietic function. In the present study, we separated and obtained this SB secretions and surprisingly found that the secretion possesses strong cytocidal activity against cultured human tumor cells. In addition, the secretion specifically destroyed the plasma membrane of breast adenocarcinoma cells (MCF-7 cells), dramatically altering their cytoskeleton and organelle morphology, which subsequently caused irreversible intracellular decay in the MCF-7 cells. The secretion possesses high cytotoxicity toward human cells and has the potential to recognize and kill several classes of cancer cells, suggesting that this secretion may have applications in medical and biological fields.

## Methods

### Animals and cell lines

This study was carried out in strict accordance with the recommendations in the Guide for the Care and Use of Laboratory Animals of the National Institutes of Health. All experiments were performed in accordance with the regulations of the Animal Welfare and Research Ethics Committee of the Institute of Dalian Medical University on Animal Care protocol (Permit Number: SYXK2004-0029). A permit from fishery administration and fishing port superintendency agency of Liaoning province Shuifeng reservoir was obtained for vertebrate study in Liaoning Province, China (Permit number: G01690). Adult lampreys (*Letheron japonicum*) (weighing 121–152 g) were bought in December 2012 from the Tongjiang Valley of Songhua River, Heilongjiang Province, China (Permit number: 033). These lampreys were maintained in glass tanks with recirculating freshwater at 10 °C at Liaoning Normal University. Before each sampling, the lampreys were anesthetized with 2-phenoxyethanol (Wako), and all efforts were made to minimize suffering.

Human colon adenocarcinoma (HT-29), human prostate carcinoma (DU145), and human cervical carcinoma (HeLa), human mammary epithelial cell (MCF-10A), human gastric epithelial cell (GES-1), human alveolar epithelial cell (L132) cell lines were gifts from Dr. Wang L M (Liaoning Academy of Agricultural Sciences, Dalian, China). Human breast adenocarcinoma (MCF-7), human caucasian chronic myelogenous leukemia (K562) and human hepatocellular carcinoma cell lines (SMMC-7721) were purchased from the ATCC (Manassas, VA, USA). The cells maintained in RPMI-1640 or DEME medium with 10 % fetal bovine serum (FBS) (Gibco, America), 1 % penicillin (100 U/mL), 1 % streptomycin (100 µg/mL) (Beyotime, China), under 5 % CO_2_ at 37 °C.

### Cell isolation and secretion preparation

The lampreys were dissected and then wiped with 70 % alcohol. The SBs and other tissues were stripped from the lampreys, and the attached muscle was carefully removed. After washing with ice-cold phosphate-buffered saline (PBS) and saturated with RPMI-1640 media supplemented with antibiotics (100 U/mL of penicillin sulfate and 100 μg/mL of streptomycin), the SBs were cut into small pieces approximately 1 × 1 mm^2^ with scissors and transferred to 25 cm^2^ cell culture flasks containing 30 mL 0.25 % trypsin solution. The culture flasks were maintained at 4 °C for 12 h. The next day, the cells released into the solution were decanted, centrifuged at 350×*g* for 5 min, and transferred to 1640 medium supplemented with 100 U/mL of penicillin sulfate and 100 μg/mL of streptomycin at 4 °C for 3 days. Then, the cells and cell secretion were separated by centrifugation, and the cell secretion was collected.

### Cytocidal activity assay via FACS

Cell death was analyzed using the Alexa Fluor^®^ 488 Annexin-V/Dead Cell kit according to the manufacturer’s instructions. The cells were harvested (~1 × 10^6^), and single-cell suspensions were incubated with lamprey SB cells or 10 μg/mL secretion for 10 or 30 min at 37 °C, and PBS was used as a negative control. Next, the cell cultures were centrifuged at 90×*g* for 5 min, and the cells were collected, washed in cold PBS, and re-centrifuged. The supernatant was discarded, and the cells were resuspended in 100 μL 1× annexin-binding buffer. Next, 5 μL Alexa Fluor^®^ 488 annexin V and 1 μL 100 μg/mL PI working solutions were added to each 100 μL of cell suspension, and the cells were incubated at room temperature for 10 min. The stained cells were analyzed by BD Biosciences FACS Canto flow cytometer, and the fluorescence was measured at emission wavelengths of 530 and 575 nm using the 488 nm excitation wavelength. The flow cytometry results were confirmed by viewing the cells under a fluorescence microscope.

### Live-cell imaging

Regarding the staining of live cells, the MCF-7 cells were grown on a confocal dish at a density of 4 × 10^4^ cells in 1.5 mL of media containing 10 % FBS, 2 mM glutamine, and 1 % Streptomycin/Penicillin. The cells were washed twice with PBS. Then, the MCF-7 cells were incubated in RPMI-1640 media containing 5 nM Hoechst (Sigma) at 37 °C for 20 min and were washed twice with PBS. Next, the cells were incubated in media containing 5 μM CellMask Deep Red plasma membrane stain (Invitrogen) at 37 °C for 3 min. Finally, after washing three times with PBS, the cells were incubated in fresh media, and the cell secretions were added to the MCF-7 cells. Live-cell imaging was executed with an Inverted microscope Zeiss LSM 780 and analyzed using Zeiss ZEN LE software.

### Transmission electron microscope (TEM) analysis

The MCF-7 cells and K562 cells were treated with 10 μg/mL of the secretion at 37 °C for 30 min, and PBS was used as a negative control. Subsequently, the cells were collected and fixed with 2.5 % glutaraldehyde solution in 100 mM sodium buffer (pH 7.2) at room temperature overnight. After washing with 100 mM sodium phosphate, the cells were further fixed with 1 % (w/v) osmium tetroxide in phosphate buffer at 4 °C for 2 h. Next, the cells were dehydrated successively in 70, 80, 90, and 100 % ethanol, transferred into propylene oxide, and embedded in Epon812. Ultrathin sections were cut with a Leica EM UC6 ultramicrotome (Germany) and mounted on a formvar-coated brass grid. The sections were stained with 2 % uranyl acetate (w/v) in 70 % methanol (v/v) and 0.5 % lead citrate. Observations and image recording of the cells were performed with a JEM-2000EX TEM.

### Scanning electron microscope (SEM) analysis

The MCF-7 cells and K562 cells were treated with 10 μg/mL cell secretion at 37 °C for 30 min, while cells treated with PBS were used as a negative control. Then, the cells were collected by centrifugation at 90×*g* for 5 min. The cells were washed with PBS, fixed with 2.5 % glutaraldehyde, and successively dehydrated in 70, 80, 90, and 100 % acetone. Subsequently, the cells were dried, mounted on aluminum stubs, and sputter coated with gold using an SBC-12 ion sputter coater. Observation and photography were performed with an SU8010 SEM.

### 3D-SIM super-resolution microscopy and image analysis

The MCF-7 cells were planted on a NEST microscope cover glass in a 12-well dish at a density of 2 × 10^4^ cells in 1 mL of RPMI-1640 media containing 10 % FBS, 2 mM glutamine, and 1 % Streptomycin/Penicillin. After the cells had seeded to the plate with uniform attachment for at least 1 day, the samples were treated with secretion at 37 °C for 20 min, while the cells treated with PBS were used as a negative control. The media were removed from the dish, and prewarmed staining solution containing 30 nM Mitotracker Red CMXROS (Invitrogen) probe was incubated with the cells at 37 °C for 20 min. The cells were washed with PBS three times, fixed with 4 % paraformaldehyde in PBS for 15 min, and washed three times with PBS. The negative control cells were treated with 0.1 % Triton X-100 for 10 min to permeabilize the membranes, and the cells were blocked with 5 % FBS at 37 °C for 2 h. Then, the cells were treated with α-tubulin antibody (1:2000 in PBS) as the primary antibody at 4 °C overnight. After washing with PBS, the cells were labeled with Alexa Fluor 488-conjugated donkey anti-mouse IgG (1:400 in PBS) as a fluorescent dye-conjugated secondary antibody at room temperature for 45 min. Then, the cells were treated with 5 μg/mL DAPI for 15 min, washed with PBS several times, and washed with H_2_O twice. Finally, the sections were mounted in Prolong Gold Antifade Reagent.

The MCF-7 cells were planted onto a confocal dish at a density of 4 × 10^4^ cells in 1.5 mL of media containing 10 % FBS, 2 mM glutamine, and 1 % Streptomycin/Penicillin and incubated overnight. The sample was incubated with 10 μg/mL secretion at 37 °C for 20 min, while the cells treated with PBS were used as a negative control. The media were removed from the culture dish and rinsed with HBSS. Next, the cells were incubated with prewarmed 1 µM ER-Tracker Green (Invitrogen) staining solution for 30 min at 37 °C. The staining solution was replaced with fresh probe-free media.

The 3D-SIM images of the MCF-7 cells were acquired on the DeltaVision OMX V3 imaging system (Applied Precision) with a 100× 1.4 oil objective (Olympus UPlanSApo), solid-state multimode lasers (488, 405, 561 nm) and electron-multiplying charge-coupled device (CCD) cameras (Evolve 512 × 512, Photometrics).

### Statistical analysis

The data are expressed as the mean ± SEM. The data were analyzed using Student’s t test and were considered statistically significant if p < 0.05.

## Results

### SB cells induce MCF-7 cell death

**Fig. 1:**
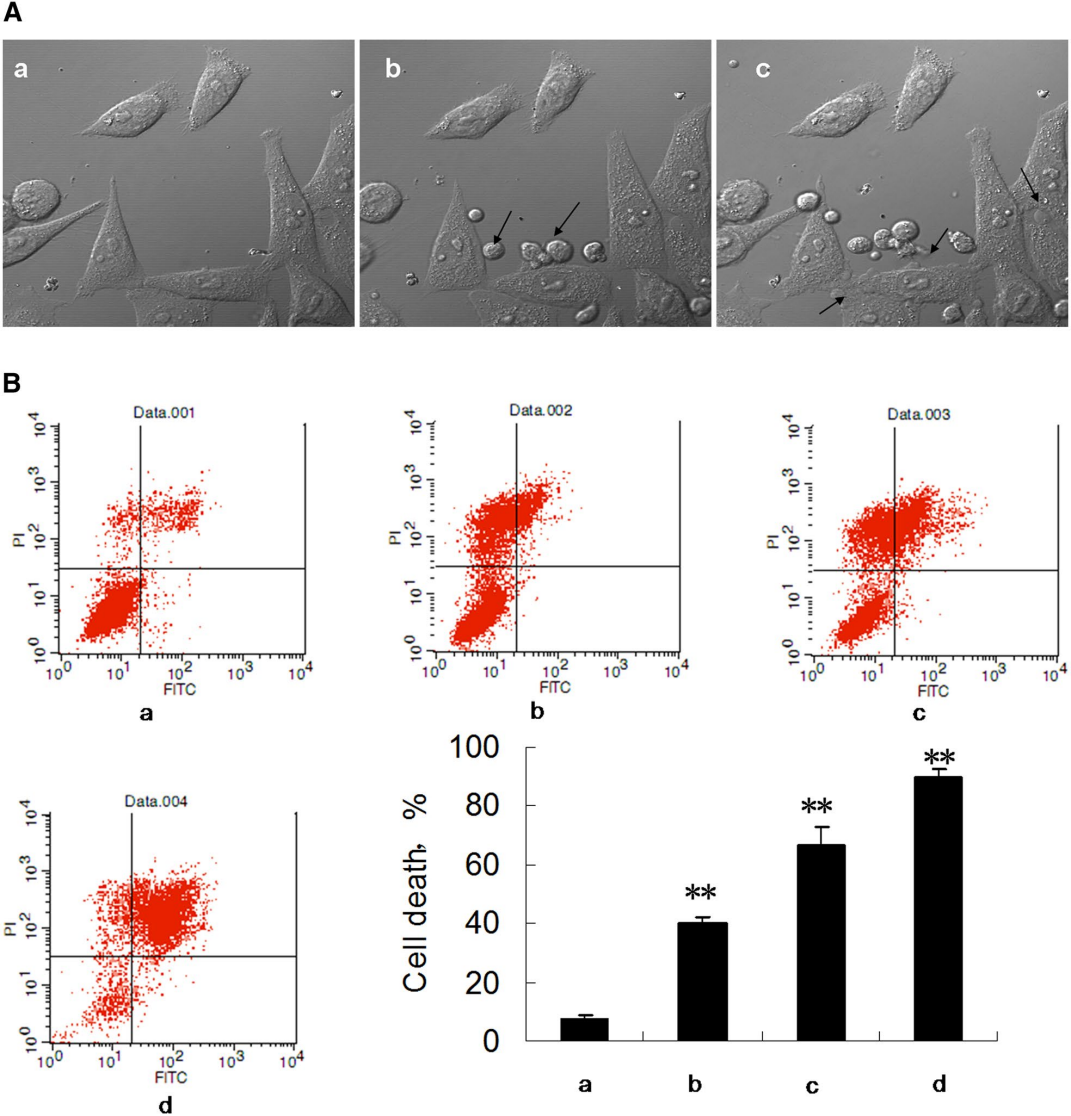
Cells from supraneural body tissues induce MCF-7 cell death. **A** Three images obtained from the video document. The MCF-7 cells were treated with an equal amount of SB cells. **B** 1 × 10^6^ MCF-7 cells were treated with various amounts of SB cells for 1 h. *1* negative control, *2* MCF-7 cell:lamprey cells 10:1, *3* MCF-7 cell:lamprey cells 5:1,* 4* MCF-7 cell:lamprey cells 1:1. Cell death was examined using the Annexin-V-FITC assay kit according to the manufacturer’s instructions and analyzed with a flow cytometer

After the live-cell imaging began, the SB cells were added to the MCF-7 cells. The SB cells surrounded the MCF-7 cells and eventually stopped at the periphery of the MCF-7 cells (Fig. 1A, panel b, see arrowheads). The outer-membranes of the MCF-7 cells were destroyed, and blebs were formed. A number of balloon-like shapes emerged one after another at the MCF-7 cell periphery (Fig. 1A, panel c, see arrowheads). Video documentation (Additional file 1: video 1) revealed the sequence of the morphological changes in the MCF-7 cells, which consisted of cell membrane blebs, intracellular vacuolations, cell swelling and finally cell bursting.

After discovering this phenomenon, we quantified the effects of SBs on the MCF-7 cells. We then incubated the two cell types for 30 min at the following different ratios: MCF-7:SB cells 1:1 (Fig. 1B, panel b), 5:1 (Fig. 1B, panel c), 10:1 (Fig. 1B, panel d) and panel a is the negative control. The ratio of MCF-7 cells death was detected by flow cytometer. The results showed that significant necrosis of MCF-7 cells can occur when incubated with SB cells in a ratio of 10:1. The proportion of necrosis increased in a dose-dependent manner for the range of the proportion (Fig. 1B, bar graph). When the MCF-7 cells:lamprey cells 1:1, the MCF-7 cell death rate even reached 90 %.

### The secretion of SB cells induces human tumor cell death

**Fig. 2:**
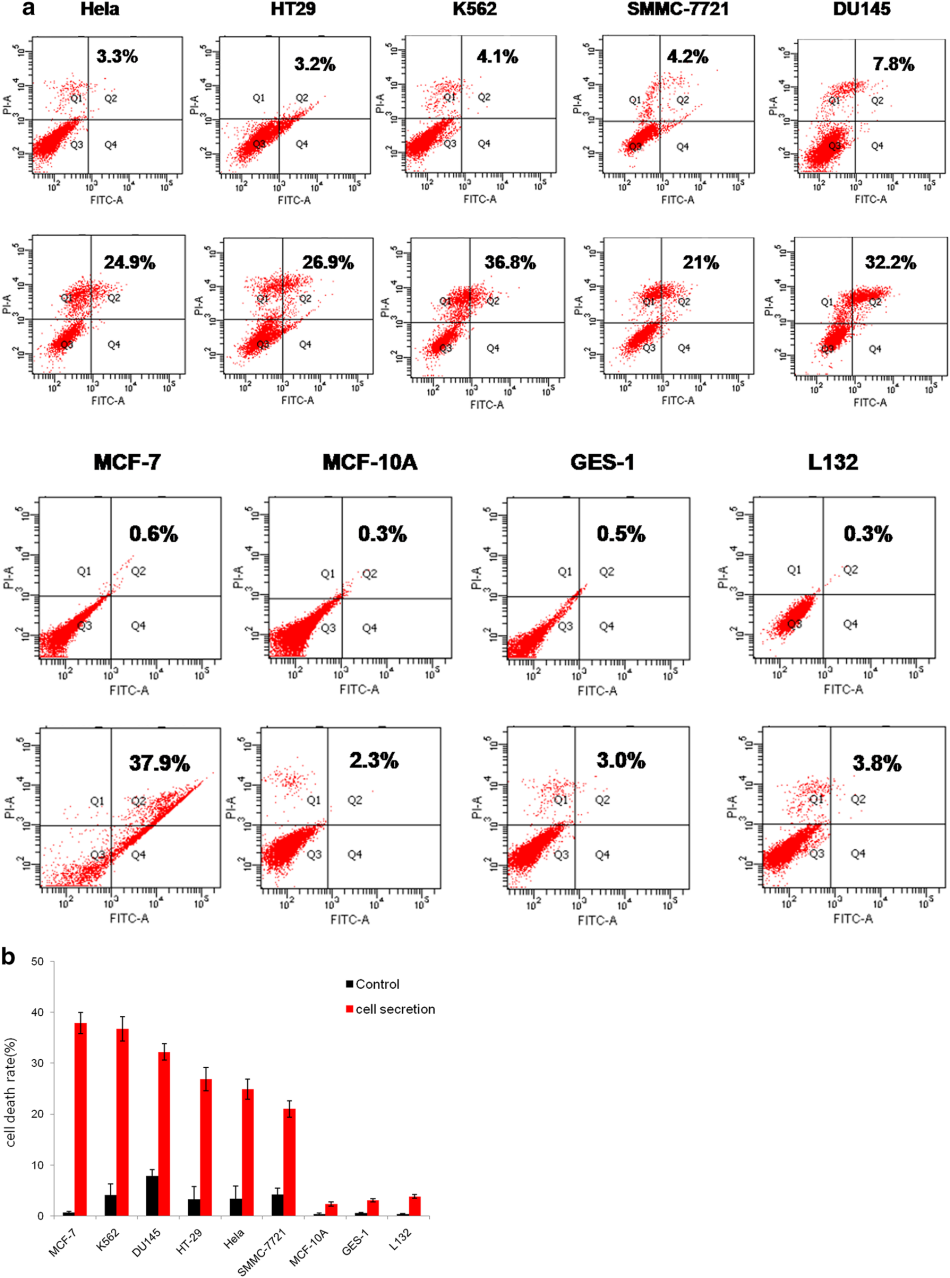
The secretion of the supraneural body tissue cells induces human tumor cell death. **a** In total, 1 × 10^6^ of different types of cells were treated with 10 µg/mL SB secretion for 10 min. Cell death was examined using the Alexa Fluor^®^ 488 Annexin-V/Dead Cell kit according to the manufacturer’s instructions and analyzed with a flow cytometer. **b** The statistical analysis of the effects of the secretion on carcinomatous cell death. Data are presented as the mean ± SEM of three independent experiments

The upper row in the Fig. 2a was the control group, which treated with the PBS, the bottom row was the secretion group. The secretion of SB cells had cytotoxicity to a number of human tumor cells including HT-29, K562, DU145, SMMC-7721, HeLa and MCF-7 cells. But the secretion had no effect on normal cells including MCF-10A, GES-1, L132. The secretion killed tumor cells at different ratios. The SMMC-7721 cell death ratio was 21 %, which was the lowest among the carcinomatous cells (Fig. 2b). The secretion had the highest cytotoxicity against the MCF-7 cells, whose death ratio reached 37.9 % (Fig. 2a). The cell death ratio of k562 was second only to MCF-7 (Fig. 2b). Accordingly, the MCF-7 and k562 cells were used in the following experiment.

### The cell secretion from different tissues induces MCF-7 cell death

**Fig. 3:**
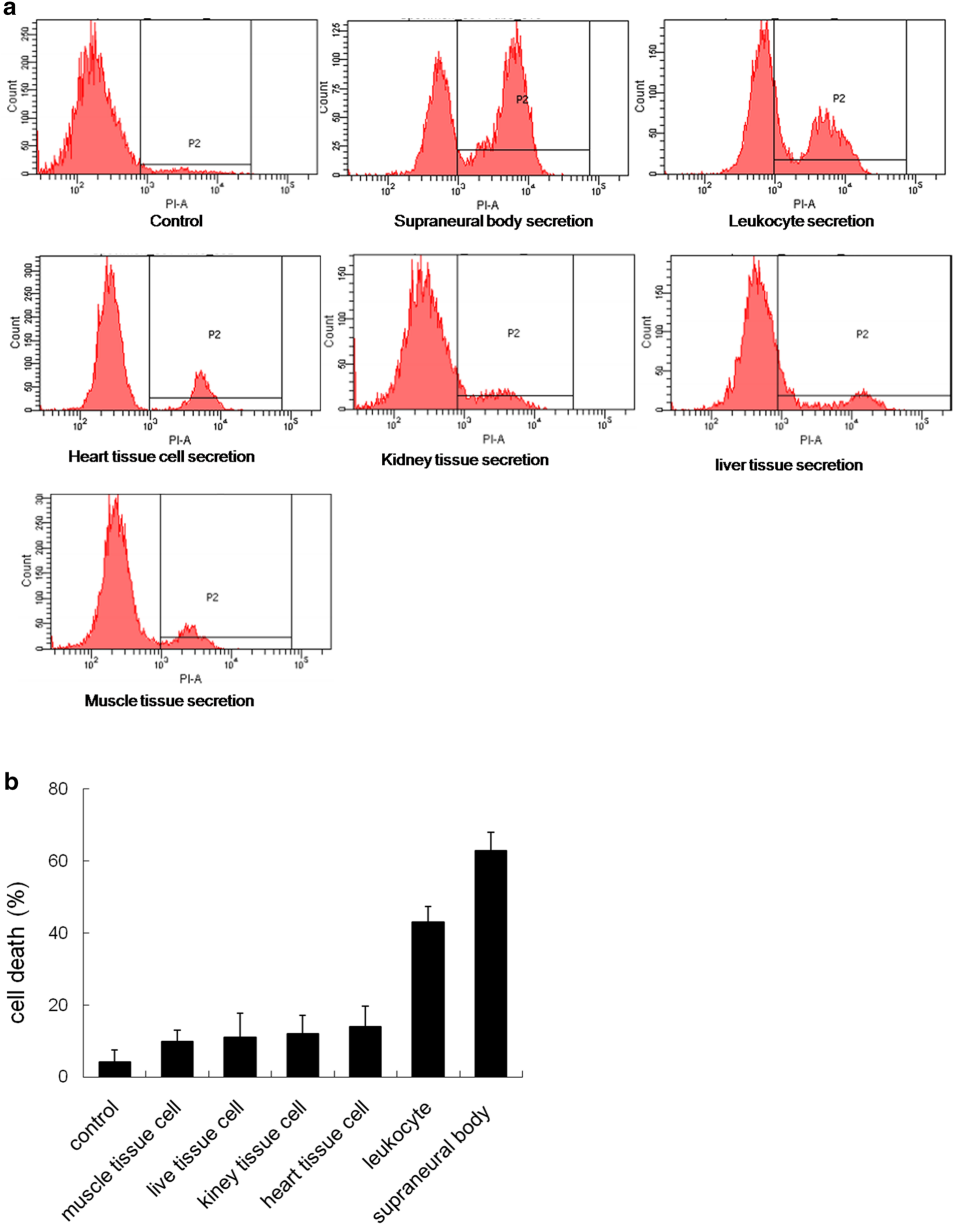
The MCF-7 cell death ratio induced by secretion of different lamprey tissue cells. **a** In total, 1 × 10^6^ MCF-7 cells were treated with 10 µg/mL of several tissue secretions for 30 min. Cell death was examined using the Alexa Fluor^®^ 488 Annexin-V/Dead Cell kit according to the manufacturer’s instructions and analyzed with a flow cytometer. **b** The statistical analysis of the effects of the secretion on MCF-7 cell death

To verify whether the secretions of other tissue cells also had a cytotoxic effect on MCF-7 cells, the cell secretion from several other lamprey tissues were separated and incubated with the MCF-7 cells. The death rate of MCF-7 was detected by flow cytometry (Fig. 3a). All six secretion types had a cytotoxic effect on the MCF-7 cells and killed the MCF-7 cells to varying degrees. Specifically, the cytotoxicity of the SB cell secretion ranked first by killing approximately 70 % of the MCF-7 cells. The leukocyte secretion had the second highest cytotoxicity, with a MCF-7 cell death ratio of 60 %. The cytotoxicity of the other four secretions was low, with the death ratio of the four groups below 20 % (Fig. 3b).

### Secretion induces morphological changes of MCF-7 and K562 cells

**Fig. 4:**
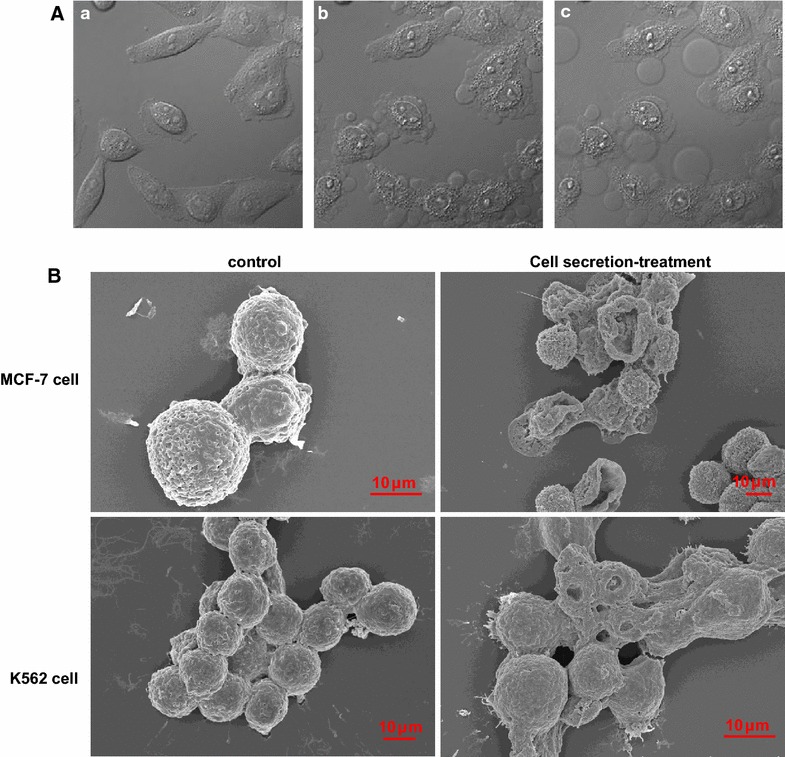
Cytopathic effects of the secretion on MCF-7 cells. **A** 5 × 10^5^ MCF-7 cells were seeded in a confocal dish, after the live-cell imaging began, the cells were treated with secretion. **B** SEM analysis of the MCF-7 and K562 cells after treatment with 10 µg/mL secretion from lamprey SB. Electron microscopy of MCF-7 or K562 cells treated for 30 min with 10 µg/mL secretion from lamprey SB cells

After 30 min of incubation with 10 µg/mL secretion, both the MCF-7 and K562 cells demonstrated dramatic morphological changes. The MCF-7 cells showed marked morphological alterations during the incubation period with the secretion. At the beginning, a number of small balloon-like shapes emerged one after another on the cell periphery in the presence of the secretion. Then, these shapes gradually enlarged over a few minutes, with several finally detaching from the cells. In addition, the outline of the cells had changed, and the intracellular particles significantly increased (Fig. 4A). Video documentation (Additional file 1: video 2) revealed the sequence of the morphological changes in the MCF-7 cells, which consisted of cell membrane blebs, intracellular vacuolations, cell swelling, and finally cell bursting.

**Fig. 5:**
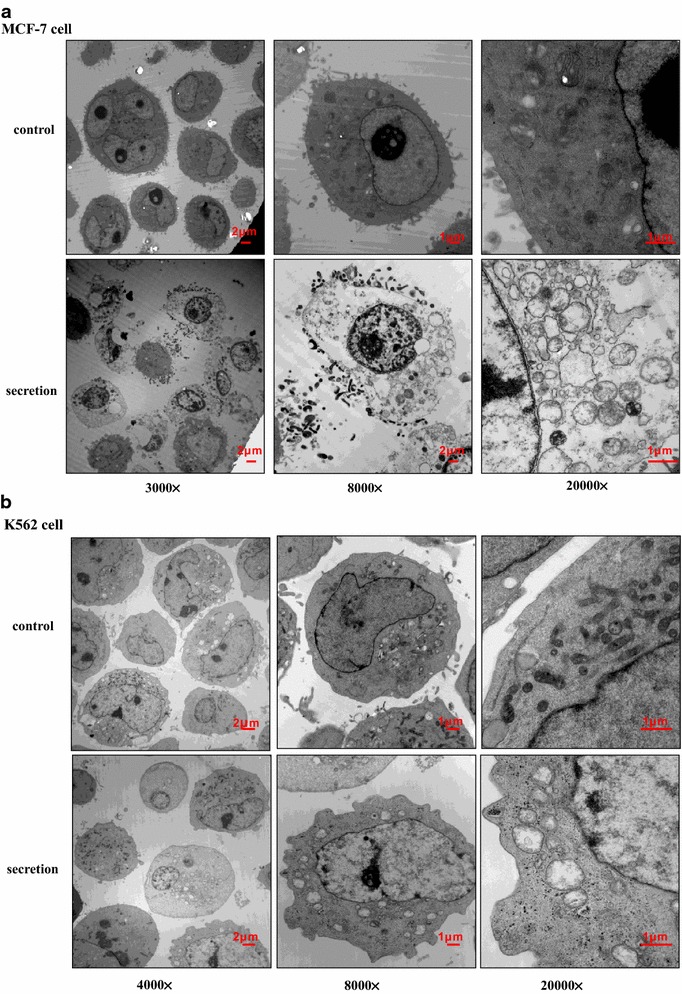
Cytopathic effects of the secretion on MCF-7 cells. **a**, **b** TEM analysis of MCF-7 and K562 cell lysis. Electron microscopy of MCF-7 or K562 cells treated for 30 min with 10 µg/mL secretion from lamprey SB cells

Morphological examination via scanning electron microscopy or transmission electron microscopy revealed that the secretion induced not only dramatic surface morphological changes but also internal changes in both the MCF-7 and K562 cells. SEM analysis of the structural morphological features showed a smooth surface with a clear globular shape, whereas cells treated with the secretion showed damage of the membrane, and several pores formed at the surface of the cells, with a diameter of the pore-like structure >1 μm (Fig. 4B). Morphological examination by TEM revealed that the secretion-treated MCF-7 and K562 cells membranes were shriveled and disrupted, and the treatment induced irreversible cytolysis, swelling of mitochondria, disruption of the crista structure, dilation of endoplasmic reticulum and finally death of the target cells. However, the untreated control cells showed an intact membrane and intact organelle structure (Fig. 5a, b).

### The MCF-7 cell plasma membranes were destroyed during the incubation with the secretion

**Fig. 6:**
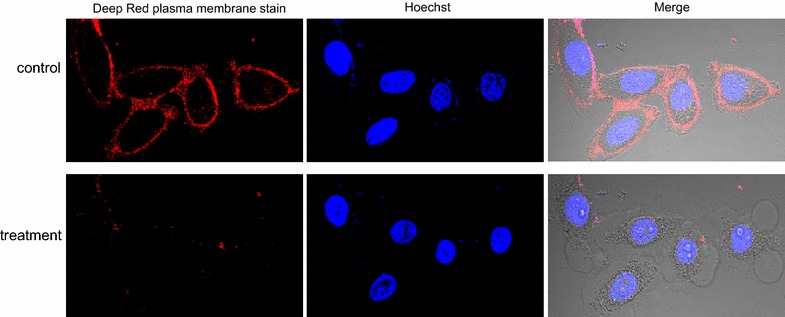
Cell plasma membrane alterations induced by secretion. In total, 5 × 10^5^ MCF-7 cells were seeded in a confocal dish. The cell nuclei and plasma membranes were stained with Hoechst and CellMask deep red, respectively. After the live-cell imaging began, the cells were treated with 10 µg/mL secretion

A number of balloon-like shapes suddenly emerged one after another on the cell periphery in the presence of 10 µg/mL secretion. One or several blebs surrounded each cell, gradually enlarged over a few minutes, and finally became detached from the cells. The plasma membranes of the cells incubated with the secretion were thoroughly destroyed, and the red fluorescence that marked the membrane was almost invisible in the secretion group. However, the intact morphology of the membrane was visible by fluorescence in secretion-untreated cells (Fig. 6).

### The secretion induces marked morphological alterations of the MCF-7 cell organelles

**Fig. 7:**
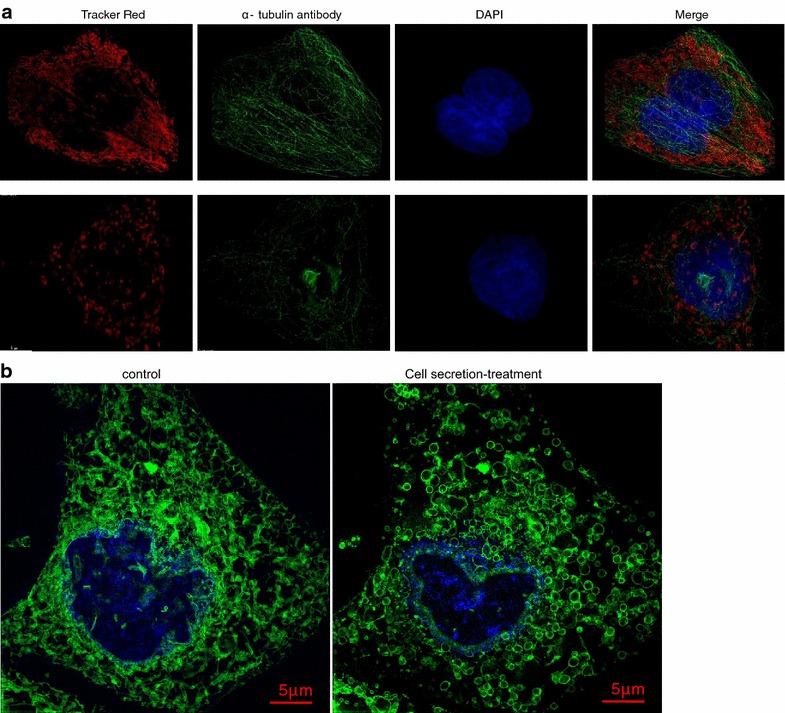
Mitochondrial, cytoskeletal and endoplasmic reticulum structure alterations induced by the secretion. **a** The alterations of the MCF-7 cell mitochondria, and cytoskeleton induced by the secretion. **b** MCF-7 cell endoplasmic reticulum structure alterations induced by secretion

The effect of the secretion on the cellular microtubule network was explored using confocal microscopy. As shown in Fig. 7a, the negative control cells exhibited normal organization of the microtubule network. The network had a continuous, long-term shape throughout the entire cell. In contrast, the secretion caused a microtubule depolymerization characterized by a destroyed filament-like structure, short microtubule fragments, and reduced microtubule density.

To elucidate whether the organelle morphologies were intact during the actions of the secretion, we observed mitochondria using the Mitotracker red probe. Extensive mitochondrial fragmentation was observed in the cells incubated with the secretion; however, the reticular morphology of the mitochondria was clearly visible by fluorescence in the negative control cells (Fig. 7a).

Next, we examined the effect of the secretion on the endoplasmic reticulum (ER) morphology and also observed marked changes. The intracellular ER network stained using ER-Tracker Green dye was converted into discontinuous fragments and vesicular or vacuolar structures by the secretion (Fig. 7b), whereas the negative control cells showed a contact, continuous network ER.

## Discussion

In previous work, the lamprey SB, also known as the protovertebral arch, possessed a dorsal fat body that appeared to be highly similar to the ‘bone marrow’ in higher vertebrates. In addition, all types of blood cells in all stages of maturity as well as their precursors are present in the tissue (Amemiya et al. 2007; Hirano et al. 2013). Adult sea lampreys were also shown to produce agglutinins, and the agglutinin-producing cells were primarily localized to the SB (Hagen et al. 1983). In the feeding adult, the SB was well developed, extending from the third gill pouch to the anterior part of the second dorsal fin. In the transverse section, the tissue was located above the nerve cord (Kirchdoerfer et al. 2012). Currently, the cytotoxic immune molecules in the lamprey have not been identified; however, Wu et al. (2013) discovered lamprey antiserum with specific exogenous cell-killing ability. This destruction process was shown to involve VLRB, C3 and C1q; however, the specific type of immune molecules directly involved in mediating the death of the target cells has not been found. Nevertheless, the question remained regarding which cells produce immune molecules. Our previous studies confirmed that the lamprey SB is an important immune tissue of the immune defense system, especially playing an important role in the adaptive immune system. The SB contains a variety of different types of cells and is rich in fat and secretes a variety of regulatory factors and other functional proteins as shown via expressed sequence tags (EST) analysis of a cDNA library from the lamprey SB.

In this study, we investigated the activation and cytocidal effects of SB secretion. The secretion induces remarkable alterations in cell morphology such as cell swelling, blebbing, and subsequent lysis (Figs. 4, 5). After MCF-7 cells were treated with the secretion, red fluorescence that labels the membrane quickly disappeared (Fig. 6), suggesting that the secretion damages the membrane structure. Considering that the secretion damages the plasma membrane and acts at this location during cytosolic leakage, the secretion may first disrupt the membrane structure by forming pores or by acting in combining with a substance that is essential to maintain stable membrane structure, which causes ions, water and protein molecules to pass through the plasma membrane at the early stage of action.

After treatment with the secretion, mitochondria swelling and fragmentation was observed (Figs. 5a, b, 7). When a cell was damaged, the most common change observed was mitochondrial swelling, which was characterized by round and bigger mitochondria, fewer cristae or even the disappearance of the mitochondria. Mitochondria are extremely sensitive to damage, and a variety of factors may lead to swelling such as hypoxia, microbial toxins, various poisons, radiation and osmotic pressure (Lu et al. 2014). Mitochondria are very complex organelles that perform a number of vital cellular functions. Their primary role is energy conversion, which results in adenosine triphosphate (ATP) production, the primary source (over 90 %) of energy for cells. Mitochondria are vital for cell life and death, cell signaling, modulation of intracellular calcium ion fluxes and mediation of cell protection. Moreover, mitochondria adapt to varying energy requirements, which change in response to energy consumption and control the production of reactive oxygen species (ROS). Mitochondria function relies on complex ion transfer processes that occur within various protein complexes present at the inner membrane (Padmaraj et al. 2014).

After treatment with the secretion, immunostaining with anti-*α*-tubulin antibodies revealed the existence of cells containing poorly organized *α*-tubulin and the remarkable disappearance of the tubulin filament structure (Fig. 7a). Microtubules are composed of *α*- and *β*-tubulin subunits, and the major function of microtubules is to partition the duplicate chromosomes equally into the daughter cells through spindle formation during mitosis (Desai and Mitchison 1997; Jordan and Wilson 2004). In malignant cells, this process is essential for tumor growth, and the dynamics of the tubulin-microtubule cycle speeds up during cell division (Jordan and Wilson 2004; Khazaei et al. 2014; Brangwynne et al. 2007; Rusan et al. 2001); therefore, microtubules are a key target for anticancer agents (Teicher 2008). Tubulin-interacting agents interfere with the dynamic equilibrium of microtubules by either inhibiting tubulin polymerization or by blocking microtubule disassembly, and both effects lead cells to cell division arrest (Bhattacharyya et al. 2008; Ravelli et al. 2004). In our experiment, the secretion damaged α-tubulin, inhibited tubulin polymerization, and blocked cell division.

The ER comprises a continuous membrane system that includes the inner and outer nuclear membranes, peripheral sheet-like structures and a network of interconnected tubules that extend into the cell periphery (Fig. 7b) (Vedrenne and Hauri 2006; Shibata et al. 2006). After incubation with the secretion, the network was converted into discontinuous fragments and vesicular or vacuolar structures (Fig. 7b). The ER plays crucial roles in the synthesis, modification, quality control and transport of integral membrane and soluble proteins destined for secretion. In animal cells, a close association of the ER occurs with the microtubule cytoskeleton, and the ER is formed along microtubules by several mechanisms (Meng et al. 2013). Increasing appreciation is developing for the close physical and functional association between mitochondria and ER (Friedman et al. 2011; Raturi and Simmen 2013; Westermann 2011). Therefore, the ER, cytoskeleton, plasma membrane, mitochondria have close connections. Thus, the secretion-damaged tumor cells may have occurred via the following two possibilities: the secretion destroyed only one organelle through mutual influence, ultimately causing the damage of other organelles or the secretion specifically destroyed membrane structure and the whole cell membrane system, which contains plasma membrane, nuclear envelope, mitochondrial outer membrane, endoplasmic reticulum membrane, was paralyzed.

In summary, the secretion induced rapid cell death in MCF-7 cells and several other tumor cells. The secretion induced remarkable cell morphological alterations such as cell blebbing. In addition, the plasma membrane was seriously destroyed by the secretion, and the secretion induced morphological alterations in the mitochondria, cytoskeletal structure and endoplasmic reticulum, eventually leading to cell death. Ultimately, the cell death process induced by the secretion was quick, violent, and irreversible. Recently, the novel protein from the secretion was purified and identified, and was confirmed involved in lamprey secretion cytotoxicity. The data was not yet published. We expect that this unique protein will allow great progress to be made in certain medical fields, such as the diagnosis and control of cancer cells.

## Additional file

10.1186/s40064-015-1270-6 Morphological change of MCF-7 cells with treatment SB cells. **Video 2.** Morphological change of MCF-7 cells with treatment SB secretion.
